# The Mitochondrial Protein C1QBP Promotes Hepatocellular Carcinoma Progression by Enhancing Cell Survival, Migration and Invasion

**DOI:** 10.7150/jca.69379

**Published:** 2022-05-09

**Authors:** Guoxin Hou, Zhimin Lu, Zhen Wang, Xinmei Yang

**Affiliations:** 1 Department of Oncology, The First Hospital of Jiaxing, Affiliated Hospital of Jiaxing University, Jiaxing, Zhejiang, China; 2 Department of outpatient, The First Hospital of Jiaxing, Affiliated Hospital of Jiaxing University, Jiaxing, Zhejiang, China; 3 Xiamen Anti-hela Biological Technology Trade Co., Ltd., Xiamen, China

**Keywords:** C1QBP, Hepatocellular carcinoma, Survival, Migration, Methylation

## Abstract

**Backgrounds:** Hepatocellular carcinoma (HCC) is a major type of death-causing cancer whose pathological mechanisms are not fully understood. In addition, the identification of effective biomarkers for HCC prognosis is in emergency. Although a variety of studies have shown that Complement C11 binding protein (C1QBP) may play a tumor-promoting or tumor-suppressive role in cancer, the functions and mechanisms of C1QBP in HCC progression are under-investigating.

**Methods and results:** Bioinformatic approaches were employed for checking the expression of *C1QBP* in HCC patient samples and the association between *C1QBP* mRNA expression and survival rates of patients with HCC or the promoter methylation of *C1QBP*. MTT analysis, PI/Annexin V staining, transwell and metabolic flux assays were performed to examine the effects of C1QBP on proliferation, apoptosis, migration, invasion, and oxidative phosphorylation of HCC cells. In the present study, we observed that *C1QBP* is lower expressed in HCC samples and cell lines. Moreover, high levels of *C1QBP* were associated with unfavorable outcomes of HCC patients. Loss-of-function assays showed that proliferation, migration and invasion of HCC cells were mitigated while cell apoptosis was augmented upon the loos of C1QBP. Moreover, the oxidative phosphorylation was moderately decreased when C1QBP was depleted. Furthermore, we also investigated the methylation status and copy number variation of *C1QBP* and analyzed their correlation with its mRNA expression in HCC patients. Finally, we suggested that *C1QBP* is correlated with genes encoding ribosome RPL-related proteins and mitochondrial MRPL-related proteins in HCC patients.

**Conclusions:** *C1QBP* is correlated with a poor prognosis of HCC patients and promotes the survival, migration and invasion of HCC cells.

## Introduction

Hepatocellular carcinoma (HCC), ranking the fourth of cancer-associated mortality, leads to a threat to increasing life expectancy globally 1. Although patients with HCC have been benefiting from the development of various effective therapeutic approaches, the five-year survival rate is still rather low (around 12% as reported in China) 2. Chronic liver diseases and Hepatitis B (HBV) infection are considered as major factors for the HCC tumorigenesis 3. These cancer-inducing factors may trigger aberrations in genomic loci encoding vital oncogenes such as *CTNNB1* (encoding β-catenin) or/and tumor suppressers including *P53* 4. Although drugs, for example a MEK inhibitor Refametinib 5, targeting key driver genes involved in HCC progression are being utilized for the clinical intervene, chemotherapy-resistance and the following cancer recurrence or metastasis still hinder the improvement of patient survival rates. Thus, unveiling the mechanisms for HCC progression is of great significance for the identification of potential therapeutic targets or agents, which might be further developed for adjuvant therapeutic strategies. Moreover, although a variety of biomarkers including *MTOR* and *RAF1* 6 have been identified for aiding to the prognosis of HCC patients, exploring biomarkers with higher effectiveness for combined prognostic application is still required.

Complement C11 binding protein (C1QBP, also named p32 and HABP1) is a multifunctional protein which plays a pivotal role in diverse cellular processes such autophagy and cell apoptosis 7, 8. It has been reported that C1QBP is mainly localized in the mitochondrial matrix and is less expressed in the cytoplasm, nucleus and on the cell surface 9-11. In addition to the functions of C1QBP in physiological processes regulation, this protein was proved to contribute to the progression of multiple cancers including breast cancers, in which the role of C1QBP were heavily investigated, renal carcinomas and pancreatic cancers 7, 12, 13. However, there are also evidence showing that C1QBP participates in the mitochondrial-dependent apoptosis, indicating C1QBP may act as a tumor suppressor 14. C1QBP was proved to be enriched in the exosomes secreted by the pancreatic cancer cells, leading to the education of the liver to form a pro-metastatic microenvironment 15. Nevertheless, the expression pattern of *C1QBP* in HCC patients and functions of C1QBP in HCC progression are not fully-investigated.

In the present study, we observed that *C1QBP* expression was upregulated in HCC patient samples and HCC cell lines. In addition, high levels of *C1QBP* were correlated with an unfavorable prognosis of patients with HCC. Loss-of-function assays showed that *C1QBP* depletion mitigated the survival, migration, invasion, and moderately attenuated the oxidative phosphorylation of HCC cells. Furthermore, the mRNA levels of *C1QBP* were demonstrated to be negatively correlated with the promoter methylation status, and to be positively correlated with copy-number alterations in genomic loci of HCC patients.

## Material and Methods

### Bioinformatic Analysis

RNA sequencing data of normal liver tissues or HCC patient tissues were downloaded from TCGA-LIHC 16 (https://portal.gdc.cancer.gov/ gov/), GSE14520 (GPL3921 Subset) 17, GSE36376 18, GSE76427 19 or ICGC-LIRI-JP 20 databases. Clinical survival data and clinical parameter information were obtained from the TCGA-LIHC, GSE76427 or ICGC-LIRI-JP databases. The promoter methylation, copy number variation (CNV) and other genomic alteration data were derived from the TCGA-LIHC, LinkedOmics (http://www.linkedomics.org/) 21 or UALCAN (http://ualcan.path.uab.edu/) 22 database. The normalization of all sequencing data and differentially expressed genes were performed by the R edgeR package (Version 3.30.3). The threshold was |log2FC (fold change)| > 1 and FDR < 0.05. The patient survival analyses were performed by the Kaplan-Meier curve R survival package (Version 3.1 12). Enrichment of *C1QBP* correlated genes was performed on the LinkedOmics platform based on data from the TCGA-LIHC database. The R package GSVA and GSEABase were used to estimate the correlation between gene expression and immune cell infiltration based on the ssGSEA algorithm 23. The R package estimate was used to check the correlation between gene expression and immune cell infiltration based on the ESTIMATE algorithm. The heatmap was generated by the pheatmap package. The p value was calculated by Log Rand test. We included all the data from indicated databases in our analysis.

### Cell Culture

HCC cell lines Li-7, SNU-182, huh-7, HCC-LM3, SMMC-7721, HepG2, MHCC-95H, PLC/PRF/5 and normal liver cell line THLE-2 were purchased from IMMOCELL (Xiamen, Fujian, China) and were maintained in Dulbecco's modified Eagle's medium (DMEM; Thermo Fisher, 41966029) supplemented with 10% fetal bovine serum (FBS; Thermo Fisher, 26140079), 100 U ml^-1^ penicillin and 100 mg ml^-1^ streptomycin (PAN Biotech). All the cells were kept in a humidified-air 5% CO_2_ atmosphere at 37°C.

### Western Blotting

Cells were lysed in RIPA buffer (Beyotime, P0013B) with freshly added 1 × complete protease inhibitor cocktail on ice and the protein concentration was determined by using a bicinchoninic acid protein assay kit (Thermo Fisher, 23225). The resulting cell lysis was subsequently boiled for 5 min at 100°C. Next, sodium dodecyl sulfate polyacrylamide gel electrophoresis (SDS-PAGE) was carried out for the separation of proteins with different molecular weight. Afterwards, the resolved proteins were transferred onto a 45-μm polyvinylidene difluoride (PVDF) membrane. A blocking buffer consists of 5% non-fat dry milk in Tris-buffered saline with 0.1% Tween 20 (TBST) was applied on the aforesaid membrane for the inhibition of non-specific binding. After 1 h incubation at room temperature (RT), the blocked membrane was subjected to appropriate primary antibodies raised against C1QBP and GAPDH that were diluted in TBST supplemented with 5% bovine serum albumin overnight at 4°C. The membranes were then washed with TBST for three times before being incubated with horseradish peroxidase (HRP)-conjugated secondary antibody raised against mouse IgG or rabbit IgG for at least 2 h at RT. Finally, the signal was detected with the aid of Clarity™ Western ECL Substrate (Thermo Fisher, 32209) and ChemiDoc Imaging System following the manufacturer's instructions. The specifications and dilutions of antibodies used in this study were listed in Supplementary Table 1.

### Real-time Quantitative PCR (RT-qPCR)

The extraction of total RNA was performed by using Trizol reagent (Invitrogen, 15596026) with standard protocol. Next, 1 μg of the isolated RNA was subjected to reverse transcription with the aid of ReverTra AceH RT Kit (Thermo Fisher, K1621). The resulting cDNAs were diluted 1:4 in nuclease-free water and 1 μl of the diluted cDNA was served as a template for real-time quantitative PCR by using SYBR Select Master Mix (Thermo Fisher, 4309155) and the primers indicated in Supplementary Table 2. The signals were captured via CFX Connect Detection System (Bio-Rad). Relative expression of *C1QBP* RNA was calculated based on the 2^-ΔΔCt^ algorithm. The levels of 18S RNA were served as an internal control for normalization. All primers used in this study were listed in Supplementary Table 2.

### Cell Viability Assay

The assessment of cell viability was done as follows. In brief, PLC/PRF/5 cells were seeded in the wells of 96-well plate at a density of 3000 cells per well. At approximately 14 h after seeding, the cells were transfected with either *C1QBP*-specifc siRNAs or non-targeting siRNA by the Lipofectamine 3000 (Thermo Fisher, L3000008) according to the manufacturer's instructions. At the indicated time points, 20 μl of 5 mg ml^-1^ 3-(4,5-Dimethylthiazol-2-yl)-2,5-diphenyltetrazolium bromide (MTT; Thermo Fisher, M6494) dissolved in phosphate-buffered saline (PBS) was directly added into each well. After 4 h incubation at 37°C, the media containing MTT were replenished with 100 μl of dimethyl sulfoxide (DMSO). Afterwards, the absorbance at OD 490 nm was immediately determined by a Microplate Reader (Bio-Rad).

### Cell Apoptosis Analysis

Flow cytometry was applied for analyzing cell apoptosis. Briefly, PLC/PRF/5 cells transfected with either *C1QBP*-specifc siRNAs or non-targeting siRNA were dissociated into single cell suspension and were sequentially stained with Annexin-V-FITC (Vazyme, Shanghai, China) and propidium iodide (PI) according to the manufacturer's suggestions. After several rounds of washes, the stained cells were directly analyzed with a BD Canto flow cytometer (BD Biosciences). Unstained cells served as negative control for gating and at least 10,000 viable single cells were acquired per sample.

### Cell Migration and Invasion Assays

The determination of cell migration capacity was initiated by plating PLC/PRF/5 cells transfected with either *C1QBP*-specifc siRNAs or non-targeting siRNA in the top chamber of 24-well transwell plates (Corning, 3422, NY, USA) containing 8 μm-pore size membrane. After 48 h incubation, cells on the lower flat were fixed, stained and photographed. In parallel, to assess cell invasion ability, the chambers containing 8 μm-pore size membrane of 24-well transwell plates were coated with Matrigel (BD Biosciences, Sparks, MD). Next, siRNA-transfected PLC/PRF/5 cells were seeded on the resulting chambers. At approximately 48 h after seeding, cells on the lower flat were fixed, stained and photographed.

### Metabolic Flux assays

Extracellular acidification rate (ECAR) and oxygen consumption rate (OCR) were quantified by an extracellular flux analyzer (XF-24, Seahorse Bioscience). At 24 h post-transfection, 5 × 10^4^ siRNA transfected PLC/PRF/5 cells were plated in each well of Seahorse XF-24 plates. The cells were pre-treated with unbuffered DMEM for 1 h. All injection reagents were adjusted to pH 7.4. Baseline rates were measured at 37°C four times before sequentially injecting the following mitochondrial inhibitors-oligomycin (10 μM), carbonycyanide p-(trifluoromethoxy) phenylhydrazone (FCCP, 1.50 μM), and rotenone (10 μM). After the addition of each inhibitor, four readings were also taken. OCR and ECAR were automatically calculated by the Seahorse XF-24 software. Each point represents an average of seven different wells.

### Statistical Analyses

GraphPad Prism version 7.0 was used for statistical analyses on the data obtained from at least three independent biological replicates. All quantitative data were presented as the mean ± standard deviation (mean ± s.d.). Statistical significances were calculated by an unpaired Student's *t*-test on the indicated dataset.* p* < 0.05 was considered statistically significant (*0.01 < *p* < 0.05, **0.001 < *p* < 0.01, ***0.0001 < *p* < 0.001, **** p < 0.0001). NS, not significant.

## Results

### *C1QBP* expression is upregulated in HCC tissues and cell lines

**Figure 1:**
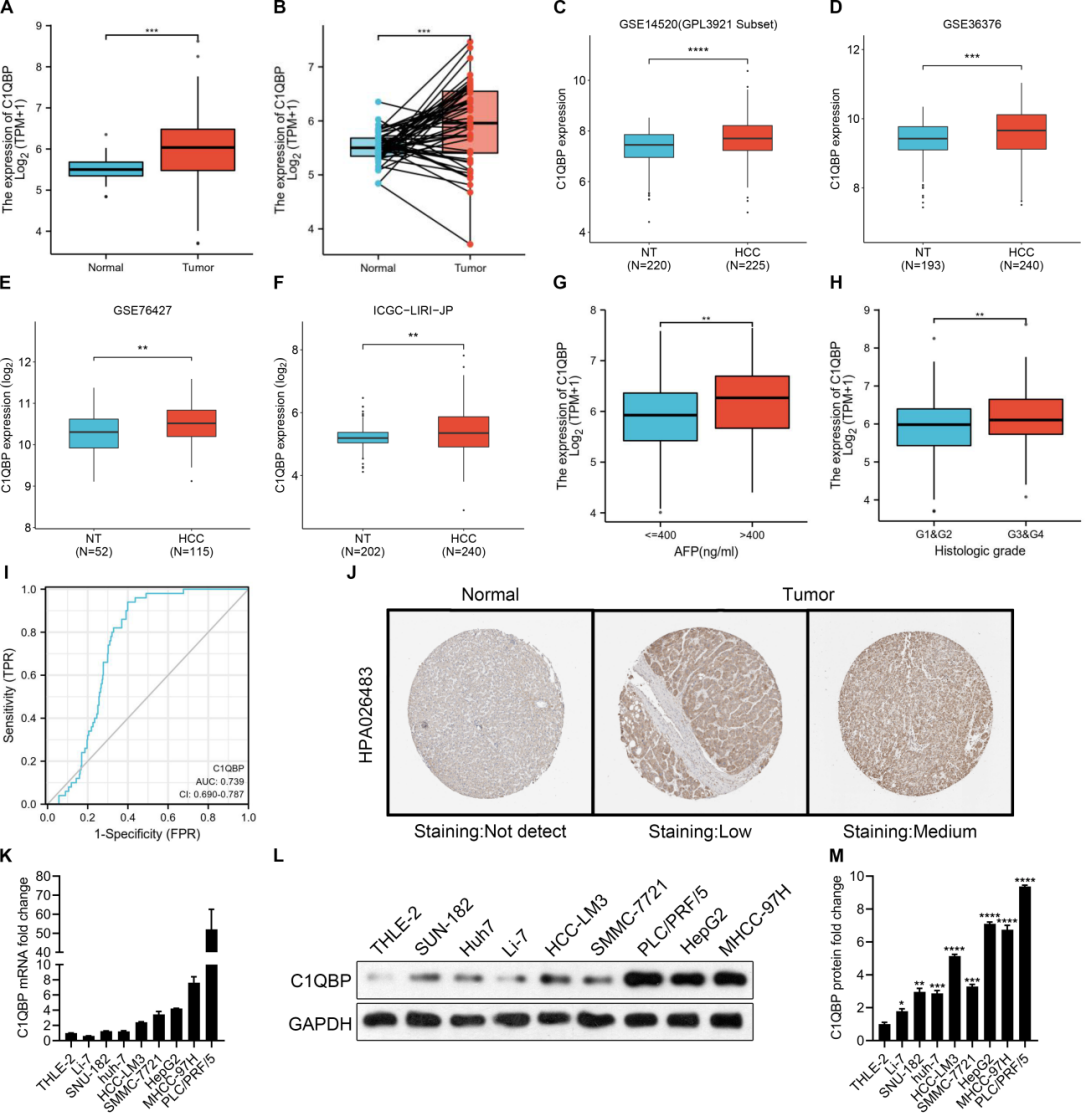
***C1QBP* is upregulated in patients with HCC and HCC cell lines.** (A, B) Unpaired (A) and paired (B, N=50) differential expression analysis of *C1QPB* between normal and tumor tissue samples from the TCGA-LIHC database. (C) Unpaired differential expression analysis of *C1QPB* between non-tumor (NT; N=220) and HCC (N=225) tissue samples from the GSE14520 dataset. (D) Unpaired differential expression analysis of *C1QPB* between NT (N=193) and HCC (N=240) tissue samples from the GSE36376 dataset. (E) Unpaired differential expression analysis of *C1QPB* between NT (N=52) and HCC (N=115) tissue samples from the GSE76427 dataset. (F) Unpaired differential expression analysis of *C1QPB* between NT (N=202) and HCC (N=240) tissue samples from the ICGC-LIRI-JP dataset. (G) Differential expression analysis of *C1QPB* between AFP low (<=400 ng/ml) and AFP high (>400 ng/ml) HCC samples from the TCGA-LIHC database. (H) Differential expression analysis of *C1QPB* between low histologic grade (G1&G2) and high histologic grades (G3&G4) HCC samples from the TCGA-LIHC database. (I) Estimated ROC curve for determining the diagnostic value (based on sensitively and specificity) of *C1QPB* in the TCGA-LIHC database. (J) Representative images of C1QBP protein expression in normal or HCC tumor samples from the Human Protein Atlas (HPA) database 36. (K) RT-qPCR quantification of *C1QBP* mRNA levels in a normal (THLE-2) liver cell line or HCC cell lines. (L, M) Western blotting analysis (L) and the quantification (M) of C1QBP protein levels in a normal (THLE-2) liver cell line or HCC cell lines. Statistical analysis was performed by comparing the indicated mRNA levels with the mRNA level in THLE-2 cells.

C1QBP, as a multicompartmental cellular protein, participates in a diversity of cellular processes, for instance, cell apoptosis, immune response, pre-mRNA splicing, mitochondrial translation and transcriptional regulation 24-28. Yet, the functions of C1QBP during the HCC development and progression have not been comprehensively defined. To this end, we started to check *C1QBP* levels in a pan-cancer manner. Based on the results from TNMplot and TIMER databases, we found that *C1QBP* was generally higher expressed in most of the cancer types including HCC (Figure S1A and 1B). Moreover, a remarkable increase of C1QBP expression was observed in HCC tumor samples compared with paired or unpaired normal tissue samples was observed in the TCGA-LIHC database and other databases (Figure 1A, Figure S2A-2C). The following comparison on data derived from paired normal and tumor tissue samples revealed a consistent trend (Figure 1B). Next, bioinformatic analyses were carried out on three datasets downloaded from the GEO database (GSE14520, GSE36376 and GSE76427) and one dataset from ICGC database, respectively, for systematically studying the differential expression of *C1QBP* in non-tumor and HCC tissue samples. In line with earlier results (Figure 1A and 1B), *C1QBP* levels were significantly higher in HCC tissue samples compared with non-tumor samples (Figure 1C-1F), indicating that *C1QBP* might be actively involved in the development and progression of HCC. In addition, increased *C1QPB* levels were detected in HCC samples with high AFP (>400 ng/ml; Figure 1G) or with high histologic grades (G3&G4; Figure 1H). Furthermore, the estimated ROC curve illustrated that the diagnostic sensitively and specificity of C1QPB were rather effective among patients with HCC (AUC=0.739; Figure 1I). The enhanced protein expression of C1QBP in HCC tumor tissues was further confirmed based on the data obtained from Human Protein Atlas (HPA) database (Figure 1J). Of note, substantially higher mRNA and protein levels of *C1QBP* were readily detected in HCC cell lines in comparison with the normal liver cells as demonstrated by RT-qPCR and western blotting analyses (Figure 1K-1M). These cumulative data indicate that *C1QBP* expression is elevated in patients with HCC and HCC cell lines, indicating it may be positively associated with the progression of HCC.

### High level of *C1QBP* is correlated with poor survival probability of HCC patients

**Figure 2:**
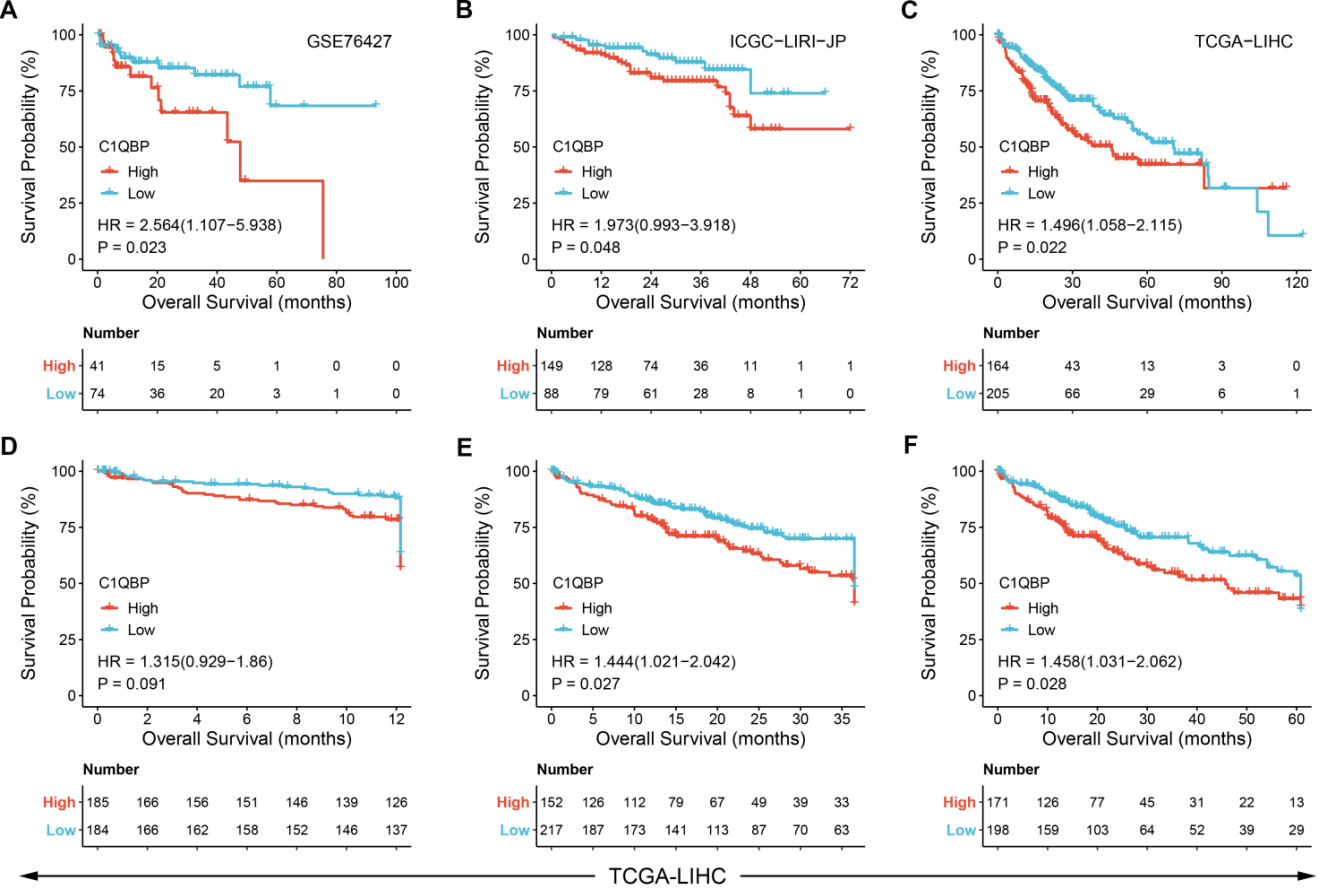
***C1QBP* is corelated with the poor prognosis in patients with HCC.** (A - C) Kaplan-Meier plots showing the survival probabilities of HCC patients stratified by *C1QBP* expression. Patient survival details were derived from GSE76427 (A), ICGC-LIRI-JP (B) or TCGA-LIHC (C) database, respectively. (D - F) Kaplan-Meier plots exhibiting the one-year (D), three-year (E) and five-year (F) survival probabilities of HCC patients stratified by *C1QBP* expression in the TCGA-LIHC database.

**Figure 3:**
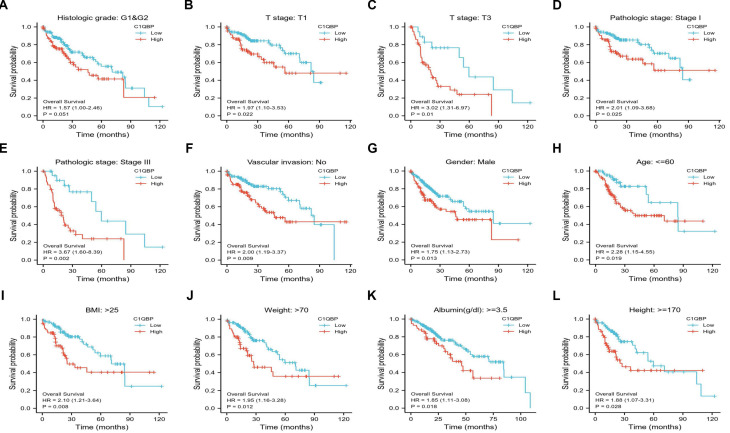
***C1QBP* is associated with unfavorable outcomes in various subgroups of patients with HCC.** Patient survival analysis of *C1QBP* in groups classified by diverse clinical parameters including histological grade (G1&G2; A), T stages (T1; B and T3; C), pathological stages (Stage I; D and Stage III; E), vascular invasion (no; F), gender (male; G), age (<=60; H), BMI (>25; I), weight (>70), albumin (>=3.5; K) and height (>=170; L). All the survival data were downloaded from the TCGA-LIHC database.

Next, we sought to identify the clinical significance of *C1QBP* in HCC patients. Therefore, publicly available datasets obtained from GEO, ICGA and TCGA databases, respectively, were exploited for overall survival probability assessment in patients diagnosed with HCC. Kaplan-Meier plots revealed that HCC patients with high *C1QBP* expression exhibited a significant poorer overall survival probability than those with low *C1QBP* levels (Figure 2A-2C, Figure S3A-3E). Likewise, a substantial decrease of survival probability was observed in HCC patients with high *C1QBP* expression based on the analyses of one-year, three-year and five-year survival probabilities of HCC patients (Figure 2D-2F). Additionally, survival probabilities were further classified by diverse clinical parameters, including histological grade (Figure 3A), T stages (Figure 3B and 3C), pathological stages (Figure 3D and 3E), vascular invasion (Figure 3F), gender (male; 3G), age (Figure 3H), BMI (Figure 3I), weight (Figure 3J), albumin (Figure 3K) and height (Figure 3L). Consistent with our previous findings, enhanced *C1QBP* expression leads to poor survival probability of HCC patients regardless of their clinical characteristics (Figure 3A-L).

### HCC cell survival, migratory and invasive abilities are hindered upon C1QBP depletion

**Figure 4:**
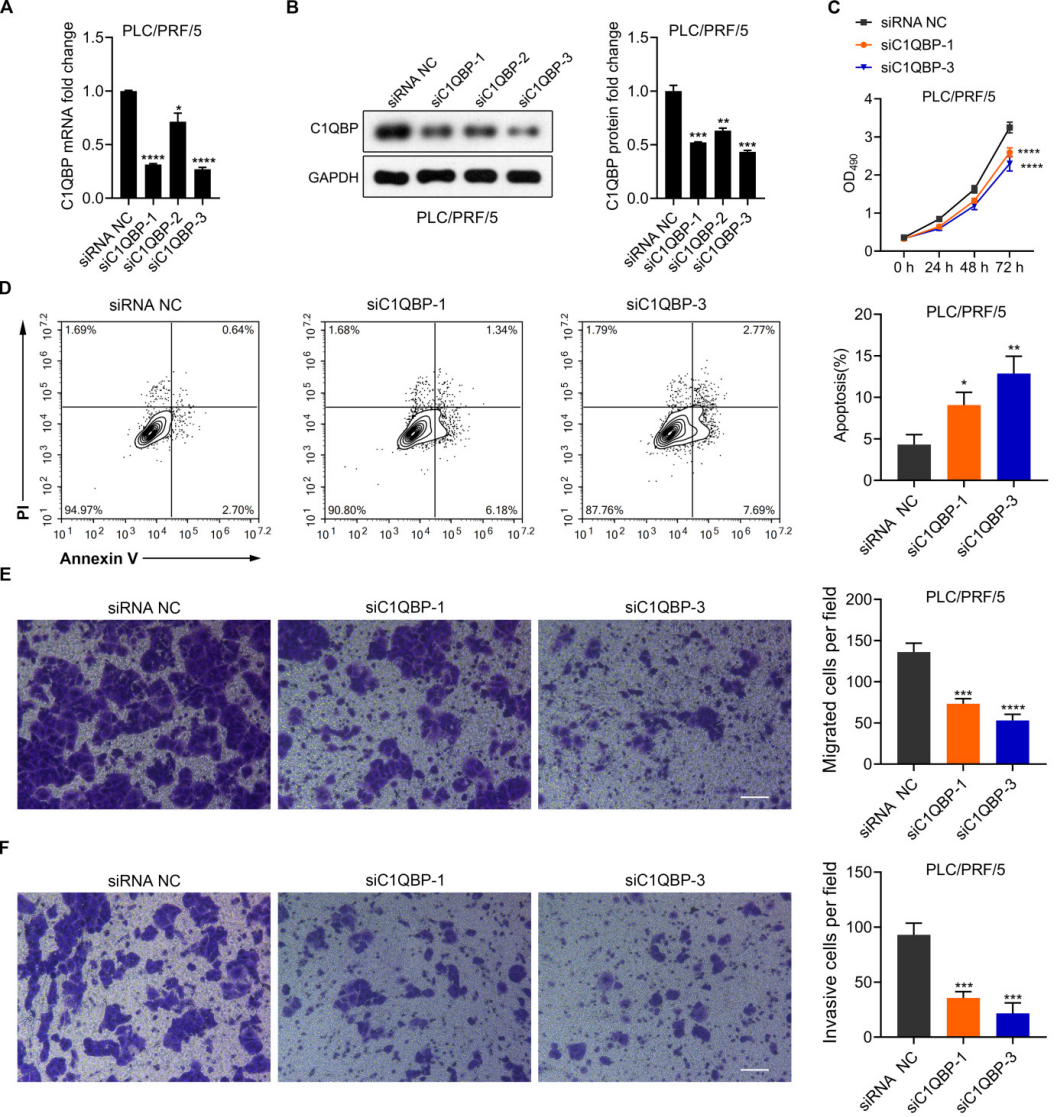
** Knockdown of C1QBP attenuates the survival, migration and invasion but augments the ATP acceleration and cell apoptosis in HCC cells.** (A) RT-qPCR detection of *C1QBP* mRNA expression in PLC/PRF/5 cells upon the transfection of three separate siRNAs against *C1QBP*. Statistical analysis was performed by comparing the indicated mRNA levels with the mRNA level from siRNA negative control (NC) cells. (B) Western blotting representative images (left) and the quantification (right) of C1QBP protein levels in PLC/PRF/5 cells upon the transfection of three separate siRNAs against *C1QBP*. Statistical analysis was performed by comparing the indicated protein levels with the protein level from siRNA NC cells. (C) MTT assays for evaluating the cell survival of PLC/PRF/5 cells upon the transfection of two separate siRNAs against *C1QBP*. Statistical analysis was performed by comparing the indicated OD490 values with the value from siRNA NC cells. (D) Flow cytometry analysis (left) and quantification (right) for checking the apoptotic abilities of PLC/PRF/5 cells transfected with two separate siRNAs against *C1QBP*. Statistical analysis was performed by comparing the indicated apoptotic percentages with the apoptotic percentages from siRNA NC cells. (E, F) Representative images and quantification results from transwell assays for assessing the migration (E) and invasion (F) of PLC/PRF/5 cells after transfecting with two siRNAs against *C1QBP*. Statistical analysis was performed by comparing the migratory/invasive cell numbers with the cell numbers from siRNA NC cells.

To further explore the functions of C1QBP in HCC cell malignancy, *C1QBP* was depleted via three distinct siRNAs specifically targeting *C1QBP* in PLC/PRF/5 cells. Significant reduction of *C1QBP* mRNA and protein was detected upon transfection of aforesaid siRNAs, as determined by RT-qPCR and western blotting analyses (Figure 4A and 4B). The most potent two siRNAs, namely siC1QBP-1 and siC1QBP-3, were selected for further investigation. We started with looking into the impact of C1QBP depletion on cell proliferation activity. Moreover, MTT assay revealed that loss of C1QBP dramatically impaired the capacity of PLC/PRF/5 cell proliferation (Figure 4C). Additional PI/Annexin V co-staining was carried out on these siRNAs transfected PLC/PRF/5 cells owing to the closely correlation between cell viability and cell apoptosis. As expected, the proportions of apoptotic cells were considerably boosted upon C1QBP depletion (Figure 4D). Of note, knocking down C1QBP in PLC/PRF/5 cell also led to the reduced cell migratory and invasive capabilities (Figure 4E and 4F). Taken together, these data suggest that C1QBP depletion attenuates the survival, migration and invasion of HCC cells.

### Loss of C1QBP reduces oxidative phosphorylation

**Figure 5:**
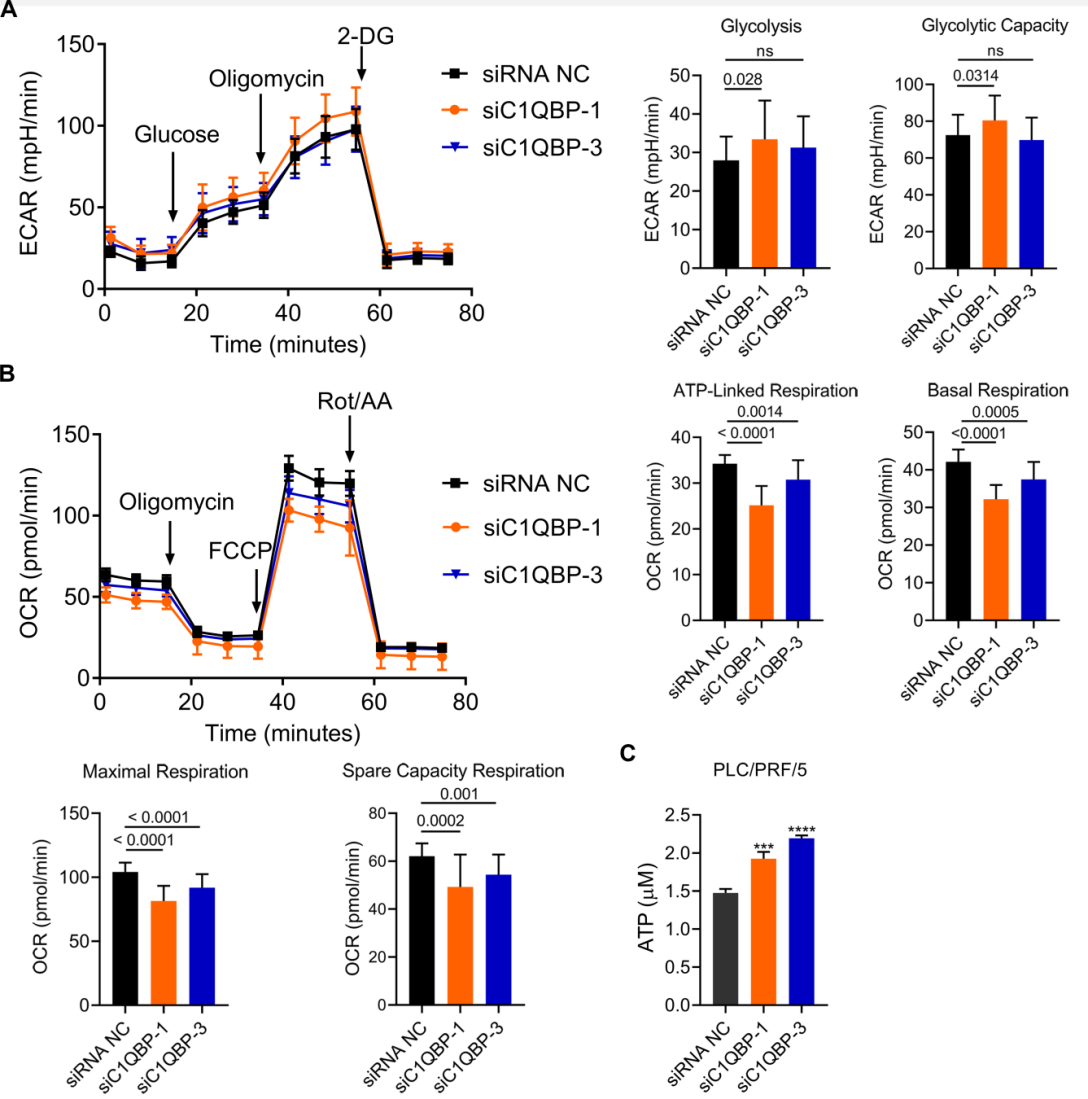
** Depletion of C1QBP decreases oxidative phosphorylation.** (A) Plots indicating the ECAR (mpH/min) determined by extracellular flux analyzer (left) and the statistical analysis of glycolysis between siC1QBP and siNC groups. After generating a baseline, Glucose, Oligomicin, and 2-DG were added sequentially. (B) Plots indicating the OCR (pmole/min) measured by extracellular flux analyzer (left) and the statistical analysis of different kinds of respirations between siC1QBP and siNC groups. After generating a baseline, Oligomycin, FCCP, and Rot/AA were added sequentially. (C) Measurement of ATP levels in PLC/PRF/5 cells upon the transfection of two separate siRNAs against C1QBP. Statistical analysis was performed by comparing the indicated ATP levels with the ATP level from siRNA NC cells.

Next, we examined whether C1QBP affects aerobic glycolysis due to its mitochondrial function in the regulation of cellular energy metabolism and oxidative phosphorylation 24-28. To test this hypothesis, extracellular acidification rate (ECAR) was determined for the examination of glycolytic flux upon knockdown of C1QBP. In this assay, the level of glycolysis was boosted in the presence of glucose, and subsequently addition of ATP synthase inhibitor oligomycin blocked oxidative phosphorylation, permitting the measurement of glycolytic capacity. Finally, 2-deoxy-D-glucose (2-DG) was added for the inhibition of glycolysis process, allowing us to evaluate the glycolytic reserve. As shown in Figure 5A, C1QBP depletion by two siRNAs led to no or minor increase on overall glycolytic flux in PLC/PRF/5 cells, including glycolysis and glycolytic capacity. In parallel, level of oxidative phosphorylation was quantified by measuring the response of oxygen consumption rate (OCR) to three well-defined modulators involved in mitochondrial respiration. The results showed that the levels of ATP-linked, basal, maximal and spare capacity respiration were significantly decreased by the knockdown of C1QBP (Figure 5B). To our surprise, the amount of ATP was also remarkably enhanced in groups receiving C1QBP-specific siRNAs when compared to non-treatment group (Figure 5C). Collectively, these data indicate that C1QBP knockdown might moderately decrease oxidative phosphorylation, yet further validation is still required.

### *C1QBP* mRNA levels are associated with the promoter methylation and copy number variation

**Figure 6:**
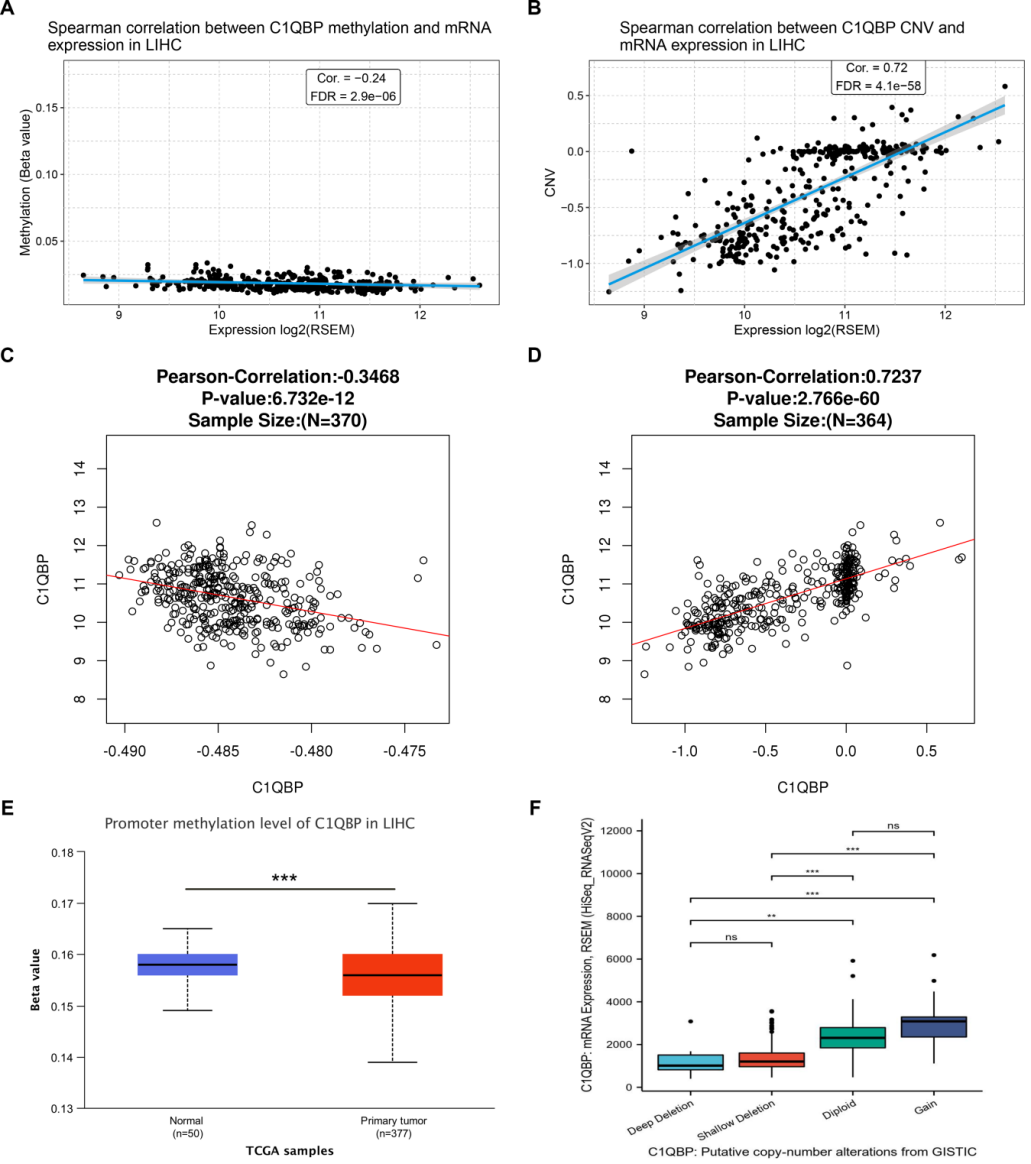
** mRNA levels of *C1QBP* is correlated with the promoter methylation and copy number variations in the genome.** (A, B) Scatter plots indicating the correlations between the expression of *C1QBP* and the methylation of promoters (A) or copy number variations (CNVs) in HCC patients from the TCGA-LIHC database. (C, D) Scatter plots indicating the correlations between the expression of *C1QBP* and the methylation of promoters (C) or CNVs in HCC patients from the LinkedOmics database. (E) Differential comparison of the *C1QBP* promoter methylation status in normal liver (n=50) or primary HCC tumor (n=377) tissues. (F) Differential comparison of the *C1QBP* mRNA levels in various indicated putative copy-number alterations in HCC tumor samples from the GISTIC database.

To gain an insight into the potential mechanism of high *C1QPB* level in HCC patients and cells, we further analyzed the correlation between *C1QPB* mRNA expression and its promoter methylation, or copy number variation (CNV), respectively, on the data obtained from the GSCA database. Interestingly, a negative correlation between *C1QPB* mRNA level and its promoter methylation was observed (Figure 6A), whereas CNV was positively associated with the *C1QPB* mRNA level (Figure 6B). Consistent with these findings, similar scenarios were detected on the data derived from LinkedOmics database (Figure 6C and 6D). In addition, promoter methylation level of *C1QPB* was significantly decreased in HCC primary tumor samples when compared with normal samples (Figure 6E). Moreover, the putative causes for copy number changes were classified into deep deletion, shallow deletion, diploid and gain, which were associated with the mRNA levels of *C1QBP* in HCC patients. In summary, these results reveal that the mRNA levels of *C1QBP* are associated with the promoter methylation and copy number alterations, and that the abnormal upregulation of *C1QPB* mRNA in HCC patients might be resulted from the low levels of its promoter methylation.

### *C1QBP* is correlated with ribosome related and mitochondrial ribosome related genes, but not associated with immune cell infiltration

**Figure 7:**
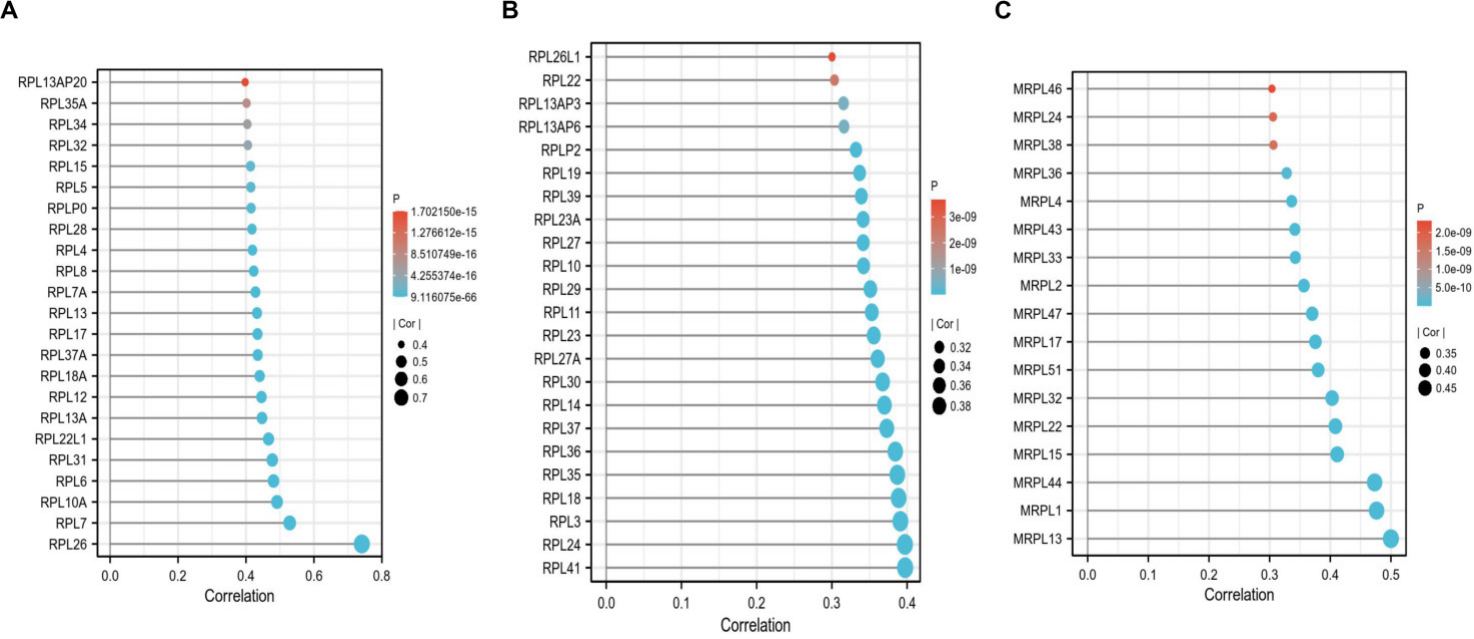
***C1QBP* is positively correlated with *RPL*-related and *MRPL*-related genes.** (A, B) Plots indicating the correlations between *C1QBP and RPL*-related genes in the TCGA-LIHC database. (C) Plots showing the correlations between *C1QBP and MRPL*-related genes in the TCGA-LIHC database. The threshold used here was |cor|>0.3 and p<0.05.

**Figure 8:**
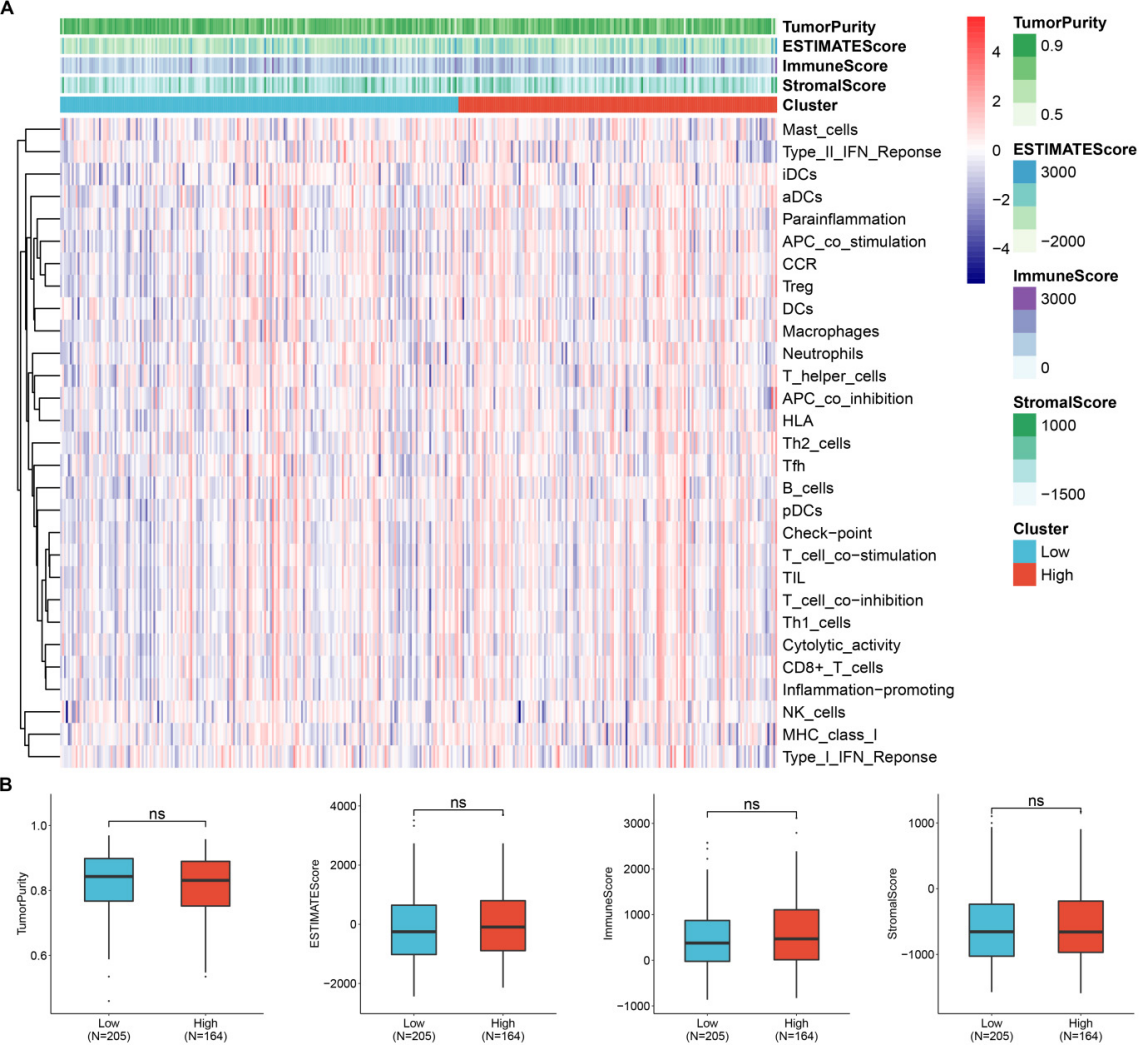
***C1QBP* expression is not associated with the infiltration of immune cells.** (A) Heatmap showing the levels of *C1QBP* mRNA (clustered as low and high) and infiltration of various immune cells. The infiltration of immune cells was calculated by ssGSEA algorithm. The expression data were from the TCGA-LIHC database. (B) Differential comparison of the TumorPurity, ESTIMATEScore, ImmuneScore or Stromalscore in HCC patients stratified by *C1QBP* mRNA levels (low, N=205; high, N=164).

To screen the co-expressed genes of *C1QBP* in patients with HCC, we performed bioinformatic analysis on the TCGA-LIHC datasets (Figure S4). Noticeably, the ribosome related genes (RPL) and mitochondrial ribosome related genes (MRPL) were enriched as genes that were highly corelated with the expression of *C1QBP* in HCC patients (Figure 7A-7C), indicating that these genes might be involved in the effects mediated by *C1QBP* on HCC cells. Importantly, GO and KEGG analysis also revealed that *C1QBP* was highly associated with the ribosome-related process (Figure S5). Considering the importance of immune cell infiltration in HCC progression 29, we evaluated the correlation between *C1QBP* expression and immune cell infiltration which was score by multiple algorithms. However, no difference was observed in HCC patents stratified by *C1QBP* expression (Figure 8A and 8B) and there were only weak to no correlations between C1QBP expression and the infiltration of immune cells or known immune genes (Figure S6 and Figure S7), suggesting that immune filtration may not be involved in the roles of *C1QBP* during HCC progression, at least based on the data from this bioinformatic quantification.

## Discussion

Since valuable prognostic markers and druggable targets with high effectiveness for HCC patients are still ill-identified, in this study we check whether *C1QBP* can serve as a biomarker for prognosis and a therapeutic target for patients with HCC. Our bioinformatic and loss-of-function studies unraveled the prognostic value of *C1QBP* for HCC patients and its suppressive effects on the viability and migration of HCC cells.

Besides the observation that *C1QBP* mRNA is more expressed in patients with HCC (Figure 1) and the high expression of *C1QBP* is correlated with a poor prognosis (Figure 2 and Figure 3), protein expression results from HPA database also revealed the trend that C1QBP protein might be higher expressed in tumor tissues compared with the normal tissues (Figure 1J). However, we did not perform further systematic analysis on the correlations between C1QBP protein levels and HCC patient survival nor the expression pattern of C1QBP in HCC tissues, due to the lack of patient databases for the evaluation of protein expression in a large scale manner. Therefore, we would like to check the protein expression of C1QBP in our own HCC patient cohort with detailed clinical parameters in the future investigation, as clinically immunohistochemical analysis on the expression of key protein markers such as Ki67 6 is widely applied for the diagnosis or/and prognosis of HCC patients.

Due to the fact that C1QBP was reported to be mainly localized in the mitochondria, the enhancing effects of C1QBP depletion on mitochondria-mediated cell apoptosis is indeed as expected (Figure 4E). However, the promotion of cell proliferation and migration/invasion of HCC cells were also observed upon the knockdown of C1QBP (Figure 4D, 4F and 4G), while ATP levels are even increased when C1QBP was knockdown (Figure 5C). Allison et al reported similar phenomenon upon the genetic manipulation of C1QBP in breast cancer cells 7. This is unexpected and the mechanism by which C1QBP negatively resulted the ATP levels in breast cancer and HCC cells need to be further studied. Moreover, the glycolysis and mitochondrial respiration were only weakly decreased upon the depletion of C1QBP (Figure 5). Thus we consider the effects of C1QBP on HCC cell proliferation and migration might not depend on the regulation of ATP levels resulting from the glycolysis and mitochondrial respiration. A possible explanation is that C1QBP might serve as a contributor to HCC progression by mitochondrial-independent mechanisms. Although we did not check the localization of C1QBP in our study, multiple studies have indicated that C1QBP is also localized in the cell surface, endoplasmic reticulum and nucleus 30-32. At the end of our study, we found that ribosome associated genes (RPL) and mitochondrial ribosome associated genes (MRPL) are enriched as genes that are highly correlated with *C1QBP* in patients with HCC (Figure 7). The correlation between *C1QBP* and these genes were found by bioinformatic method in a HCC patient online database TCGA-LIHC. Therefore, validating these correlations in a large panel of HCC samples by western blotting or RT-qPCR in the future investigation is required. In addition, we hypothesize that the ribosome-related genes might be involved in the effects directed by C1QBP on cell proliferation and migration/invasion. Thus, genetic manipulation of these genes can be further performed to pursue whether they participate in this case.

Moreover, we could not rule out the possibility that the weak effects on glycolysis and respiration are resulted from the incomplete depletion of *C1QBP*, despite the knockdown efficacy is as high as 70% by two siRNAs (Figure 4A). Therefore, CRISPR (clustered regularly interspaced short palindromic repeat)-mediated gene knockout 33 strategy can be utilized to fully silence the expression of C1QBP protein, thereby better unveiling the changes at the cellular and molecular levels. In addition, complementing with gain-of-functional analysis using the ectopic expression system or the recently developed CRISPR activation (CRISPRa) 34 platform in the further study would better illustrate the roles of C1QBP in promoting HCC cell malignancy. Moreover, to better convey broad applicability, our results can be further validated in other HCC cell lines in future study.

We also suggested that the mRNA expression *C1QBP* is corelated with the methylation of its promoter and the genetic copy number changes, and that the methylation of *C1QBP* promoter is downregulated in HCC patients (Figure 6). Further exploring the methylation level of promoter region in *C1QBP* by in methylation sequencing or CHIP-seq data in cell lines or patient samples may help to examine the promoter methylation patter of *C1QBP* in detail. In addition, it is of significance to investigate mechanisms that contribute to these genetic or epigenetic alterations of *C1QBP*, which may aid to pharmaceutically modulate the expression of *C1QBP* or identify novel biomarkers for HCC patients. Moreover, investigating the correlation between these changes and patient prognosis in other cohorts is required for evaluating their prognostic value.

In the last part of this study, we checked the association between *C1QBP* and immune cell infiltration (Figure 8), as the dysregulation of immune-suppression or immune cells infiltration results in cancer progression 35. Since the results are generated from analyzing the patient datasets, *in vivo* models with the absence of *C1QBP* might be applied to systematically study whether the immune cell infiltration is altered in *in vivo* models.

## Supplementary Material

Supplementary figures.

## Abbreviations

C1QBP: Complement C11 binding protein
HCC: hepatocellular carcinoma
HBV: Hepatitis B
CNV: copy number variation
DMEM: Dulbecco's modified Eagle's medium
FBS: fetal bovine serum
SDS-PAGE: sodium dodecyl sulfate polyacrylamide gel electrophoresis
PVDF: polyvinylidene difluoride
TBST: Tris-buffered saline with 0.1% Tween 20
RT: room temperature
HRP: horseradish peroxidase
PBS: phosphate-buffered saline
DMSO: dimethyl sulfoxide
ECAR: Extracellular acidification rate
OCR: oxygen consumption rate
FCCP: carbonycyanide p-(trifluoromethoxy) phenylhydrazone
HPA: Human Protein Atlas
MRPL: mitochondrial ribosome related genes
RPL: ribosome associated genes
CRISPR: clustered regularly interspaced short palindromic repeat
CRISPRa: CRISPR activation

## References

[1] Villanueva A (2019). Hepatocellular Carcinoma. New Engl J Med.

[2] Zheng R, Qu C, Zhang S, Zeng H, Sun K, Gu X (2018). Liver cancer incidence and mortality in China: Temporal trends and projections to 2030. Chinese journal of cancer research = Chung-kuo yen cheng yen chiu.

[3] Hayashi S, Nagaoka K, Tanaka Y (2021). Blood-Based Biomarkers in Hepatitis B Virus-Related Hepatocellular Carcinoma, Including the Viral Genome and Glycosylated Proteins. International journal of molecular sciences.

[4] Yim SY, Lee JS (2021). An Overview of the Genomic Characterization of Hepatocellular Carcinoma. J Hepatocell Carcinoma.

[5] Das M (2018). Refametinib in RAS-mutated hepatocellular cancer. The Lancet Oncology.

[6] Moldogazieva NT, Zavadskiy SP, Sologova SS, Mokhosoev IM, Terentiev AA (2021). Predictive biomarkers for systemic therapy of hepatocellular carcinoma. Expert Rev Mol Diagn.

[7] McGee AM, Douglas DL, Liang Y, Hyder SM, Baines CP (2011). The mitochondrial protein C1qbp promotes cell proliferation, migration and resistance to cell death. Cell cycle.

[8] Jiao H, You H (2016). p32: A new player in autophagy. Mol Cell Oncol.

[9] Majumdar M, Meenakshi J, Goswami SK, Datta K (2002). Hyaluronan binding protein 1 (HABP1)/C1QBP/p32 is an endogenous substrate for MAP kinase and is translocated to the nucleus upon mitogenic stimulation. Biochemical and biophysical research communications.

[10] McGee AM, Baines CP (2011). Complement 1q-binding protein inhibits the mitochondrial permeability transition pore and protects against oxidative stress-induced death. The Biochemical journal.

[11] van Leeuwen HC, O'Hare P (2001). Retargeting of the mitochondrial protein p32/gC1Qr to a cytoplasmic compartment and the cell surface. J Cell Sci.

[12] Wang Y, Fu D, Su J, Chen Y, Qi C, Sun Y (2017). C1QBP suppresses cell adhesion and metastasis of renal carcinoma cells. Sci Rep.

[13] Shi H, Fang W, Liu M, Fu D (2017). Complement component 1, q subcomponent binding protein (C1QBP) in lipid rafts mediates hepatic metastasis of pancreatic cancer by regulating IGF-1/IGF-1R signaling. International journal of cancer Journal international du cancer.

[14] Chowdhury AR, Ghosh I, Datta K (2008). Excessive reactive oxygen species induces apoptosis in fibroblasts: role of mitochondrially accumulated hyaluronic acid binding protein 1 (HABP1/p32/gC1qR). Experimental cell research.

[15] Xie Z, Gao Y, Ho C, Li L, Jin C, Wang X (2021). Exosome-delivered CD44v6/C1QBP complex drives pancreatic cancer liver metastasis by promoting fibrotic liver microenvironment. Gut.

[16] Cancer Genome Atlas Research Network (2017). Electronic address wbe, Cancer Genome Atlas Research N. Comprehensive and Integrative Genomic Characterization of Hepatocellular Carcinoma. Cell.

[17] Roessler S, Jia HL, Budhu A, Forgues M, Ye QH, Lee JS (2010). A unique metastasis gene signature enables prediction of tumor relapse in early-stage hepatocellular carcinoma patients. Cancer Res.

[18] Lim HY, Sohn I, Deng S, Lee J, Jung SH, Mao M (2013). Prediction of disease-free survival in hepatocellular carcinoma by gene expression profiling. Annals of surgical oncology.

[19] Grinchuk OV, Yenamandra SP, Iyer R, Singh M, Lee HK, Lim KH (2018). Tumor-adjacent tissue co-expression profile analysis reveals pro-oncogenic ribosomal gene signature for prognosis of resectable hepatocellular carcinoma. Molecular oncology.

[20] Zhang J, Bajari R, Andric D, Gerthoffert F, Lepsa A, Nahal-Bose H (2019). The International Cancer Genome Consortium Data Portal. Nature biotechnology.

[21] Vasaikar SV, Straub P, Wang J, Zhang B (2018). LinkedOmics: analyzing multi-omics data within and across 32 cancer types. Nucleic Acids Research.

[22] Chandrashekar DS, Bashel B, Balasubramanya SAH, Creighton CJ, Ponce-Rodriguez I, Chakravarthi B (2017). UALCAN: A Portal for Facilitating Tumor Subgroup Gene Expression and Survival Analyses. Neoplasia.

[23] Charoentong P, Finotello F, Angelova M, Mayer C, Efremova M, Rieder D (2017). Pan-cancer Immunogenomic Analyses Reveal Genotype-Immunophenotype Relationships and Predictors of Response to Checkpoint Blockade. Cell Rep.

[24] Ghebrehiwet B, Peerschke EIB (2004). CClq-R (calreticulin) and gClq-R/p33: ubiquitously expressed multi-ligand binding cellular proteins involved in inflammation and infection. Molecular Immunology.

[25] Itahana K, Zhang YP (2008). Mitochondrial p32 is a critical mediator of ARF-induced apoptosis. Cancer cell.

[26] Jiang JZ, Zhang Y, Krainer AR, Xu RM (1999). Crystal structure of human p32, a doughnut-shaped acidic mitochondrial matrix protein. Proceedings of the National Academy of Sciences of the United States of America.

[27] Xu LJ, Xiao NM, Liu F, Ren HW, Gu J (2009). Inhibition of RIG-I and MDA5-dependent antiviral response by gC1qR at mitochondria. Proceedings of the National Academy of Sciences of the United States of America.

[28] Yagi M, Uchiumi T, Takazaki S, Okuno B, Nomura M, Yoshida S (2012). p32/gC1qR is indispensable for fetal development and mitochondrial translation: importance of its RNA-binding ability. Nucleic Acids Research.

[29] Lawal G, Xiao Y, Rahnemai-Azar AA, Tsilimigras DI, Kuang M, Bakopoulos A (2021). The Immunology of Hepatocellular Carcinoma. Vaccines (Basel).

[30] Fogal V, Zhang L, Krajewski S, Ruoslahti E (2008). Mitochondrial/cell-surface protein p32/gC1qR as a molecular target in tumor cells and tumor stroma. Cancer Research.

[31] van Leeuwen HC, O'Hare P (2001). Retargeting of the mitochondrial protein p32/gC1Qr to a cytoplasmic compartment and the cell surface. Journal of Cell Science.

[32] Chattopadhyay C, Hawke D, Ryuji KI, Maity SN (2004). Human p32, interacts with B subunit of the CCAAT-binding factor, CBF/NF-Y, and inhibits CBF-mediated transcription activation *in vitro*. Nucleic Acids Research.

[33] Ran FA, Hsu PD, Wright J, Agarwala V, Scott DA, Zhang F (2013). Genome engineering using the CRISPR-Cas9 system. Nat Protoc.

[34] Konermann S, Brigham MD, Trevino AE, Joung J, Abudayyeh OO, Barcena C (2015). Genome-scale transcriptional activation by an engineered CRISPR-Cas9 complex. Nature.

[35] Giraud J, Chalopin D, Blanc JF, Saleh M (2021). Hepatocellular Carcinoma Immune Landscape and the Potential of Immunotherapies. Front Immunol.

[36] Uhlen M, Fagerberg L, Hallstrom BM, Lindskog C, Oksvold P, Mardinoglu A (2015). Proteomics. Tissue-based map of the human proteome. Science.

