# Supplementary material for: The Mitochondrial Protein C1QBP Promotes Hepatocellular Carcinoma Progression by Enhancing Cell Survival, Migration and Invasion
Source: J Cancer. 2022 May 9;13(8):2477–89. doi: 10.7150/jca.69379 (PMC9174857; doi:10.7150/jca.69379)
Supplement: Supplementary file 1 — Supplementary figures. [file jcav13p2477s1.pdf]

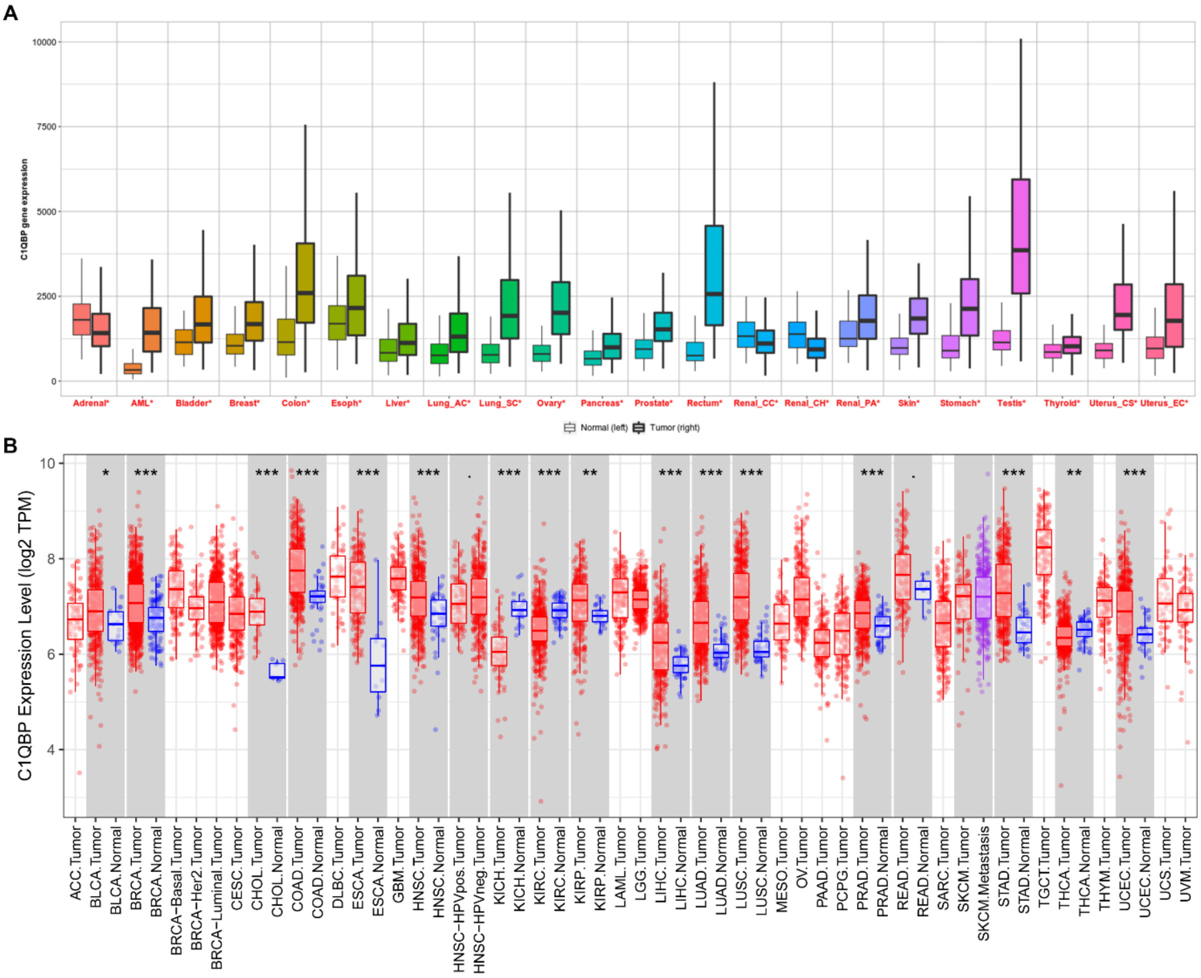

Figure S1. Pan-cancer analysis of *C1QBP* expression

(A) Plot showing the expression of *C1QBP* in normal (left) or tumor (right) samples from multiple cancer types. The figure was generated based on the transcriptomic data from the TNMplot database (https://tnmplot.com/analysis/). (B) Plot indicating *C1QBP* levels in normal and tumor samples. The figure was generated on the transcriptomic data from the TIMER database (https://cistrome.shinyapps.io/timer/).

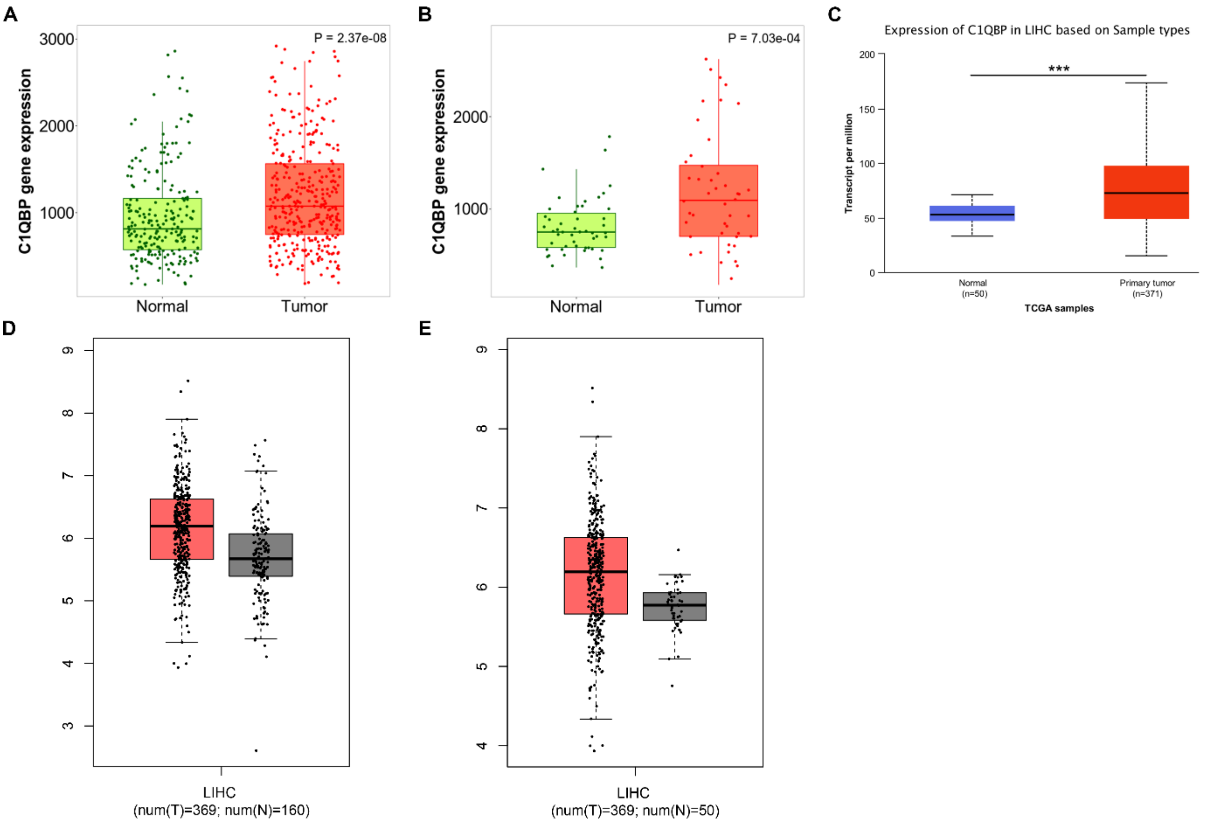

Figure S2. Differential expression analysis of *C1QBP* in diverse databases

(A-E) Differential expression analysis of *C1QBP* between normal (N) and HCC tumor samples (T) in the TNMplot database including all the samples (A) or only paired samples (B), in the UALCAN database (http://ualcan.path.uab.edu/; C) and the GEPIA2 database (http://gepia2.cancer-pku.cn/) including all the samples (D) or without the GTEx normal samples (E).

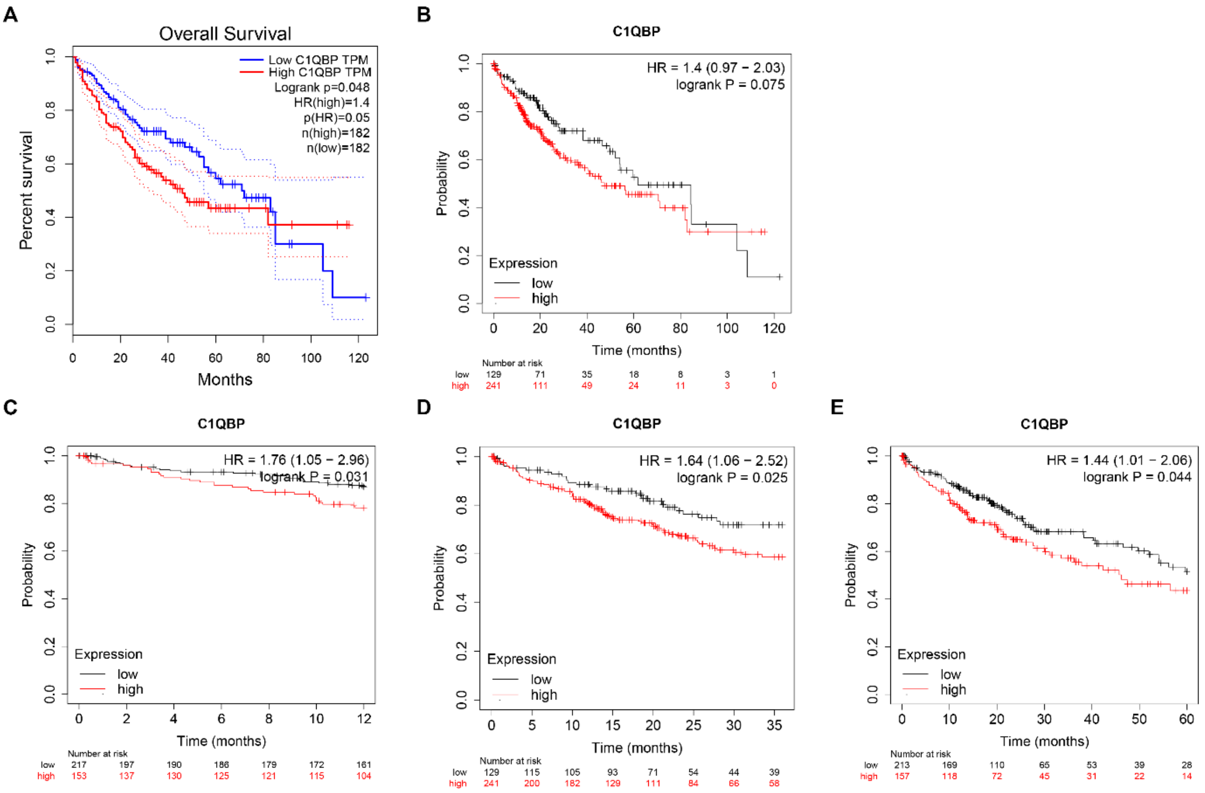

Figure S3. Survival analysis of *C1QBP* in databases

(A) Overall survival analysis of HCC patients stratified by *C1QBP* expression in the GEPIA2 database. (B-E) Overall survival analysis (B), 1-year survival analysis (C), 3-year survival analysis (D) or 5-year survival analysis (E) of HCC patients stratified by *C1QBP* expression in the Kaplan-Meier Plotter database.

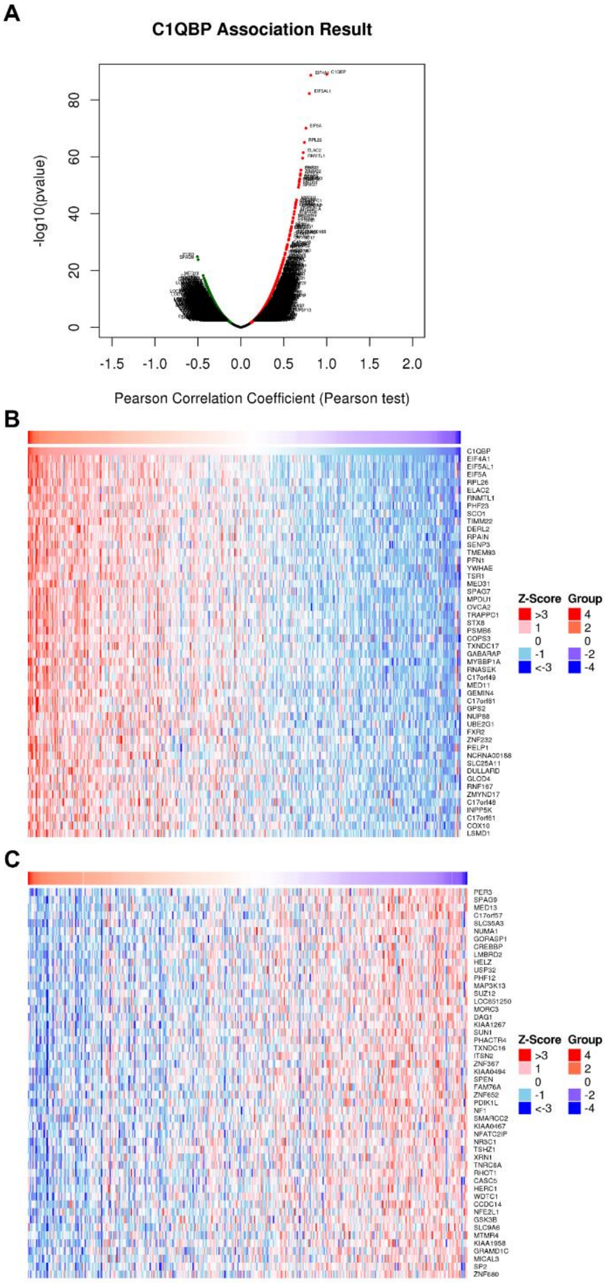

Figure S4. Enrichment of *C1QBP* associated genes

(A) Volcano plot showing the associated genes of *C1QBP*. (B, C) Heat map showing the positively (B) and negatively (C) corelated genes of *C1*QBP. All the associations were analyzed based on the TCGA-LIHC database using the LinkedOmics platform (http://www.linkedomics.org/).

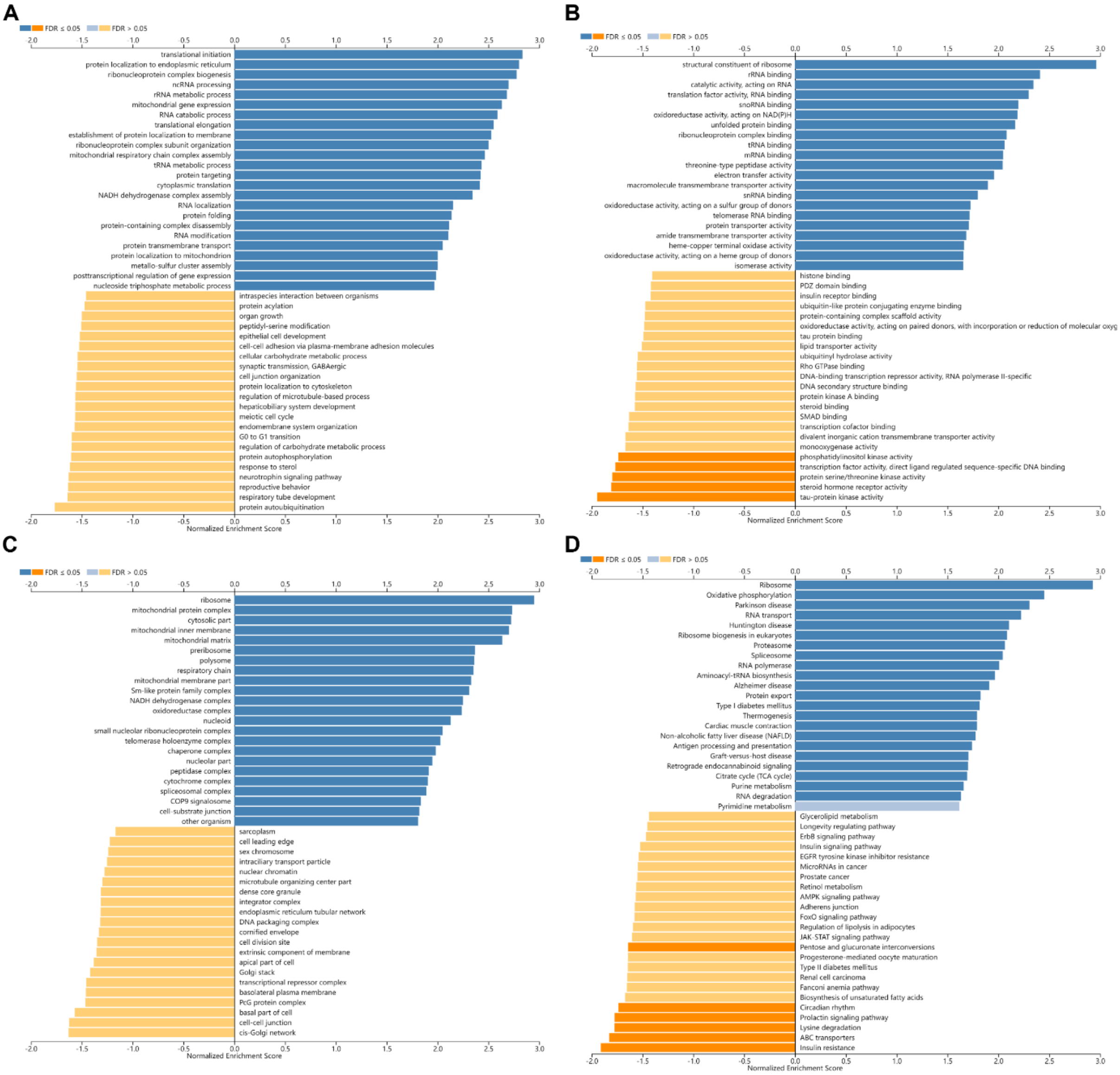

Figure S5. GO and KEGG analysis of *C1QBP* associated processes and pathways

(A-C) GO analysis of *C1QBP* correlated biological process (BP; A), molecular function (MF; B) and cellular component (CC; C). (D) KEGG analysis of *C1QBP* correlated biological pathways.

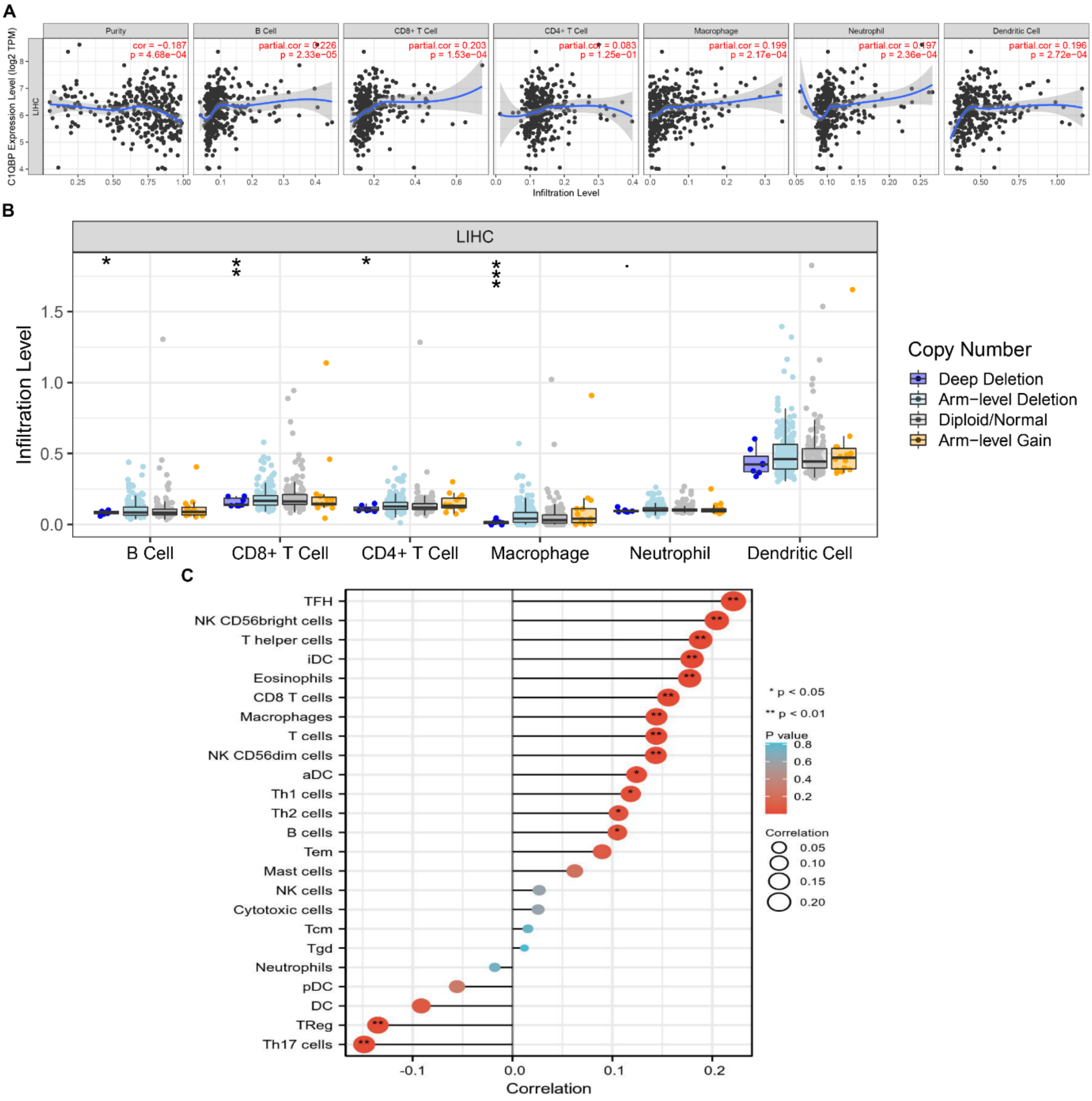

Figure S6. Correlation between *C1QBP* expression and immune cell infiltration

(A) Plots showing the levels of *C1QBP* mRNA and infiltration of various immune cells. (B) Plots indicating the copy number variance of *C1QBP* gene and infiltration of various immune cells. The expression data for Figure A and B were derived from the TCGA-LIHC database and analyzed by the TIMER platform (https://cistrome.shinyapps.io/timer/). (C) Forest plot showing the correlation between *C1QBP* expression and 24 immune cells. Immune infiltration analysis was performed by single-sample gene set enrichment analysis (ssGSEA) in the “GSVA” R package, and the infiltration levels of 24 immune cell types were quantified from gene expression profiles.

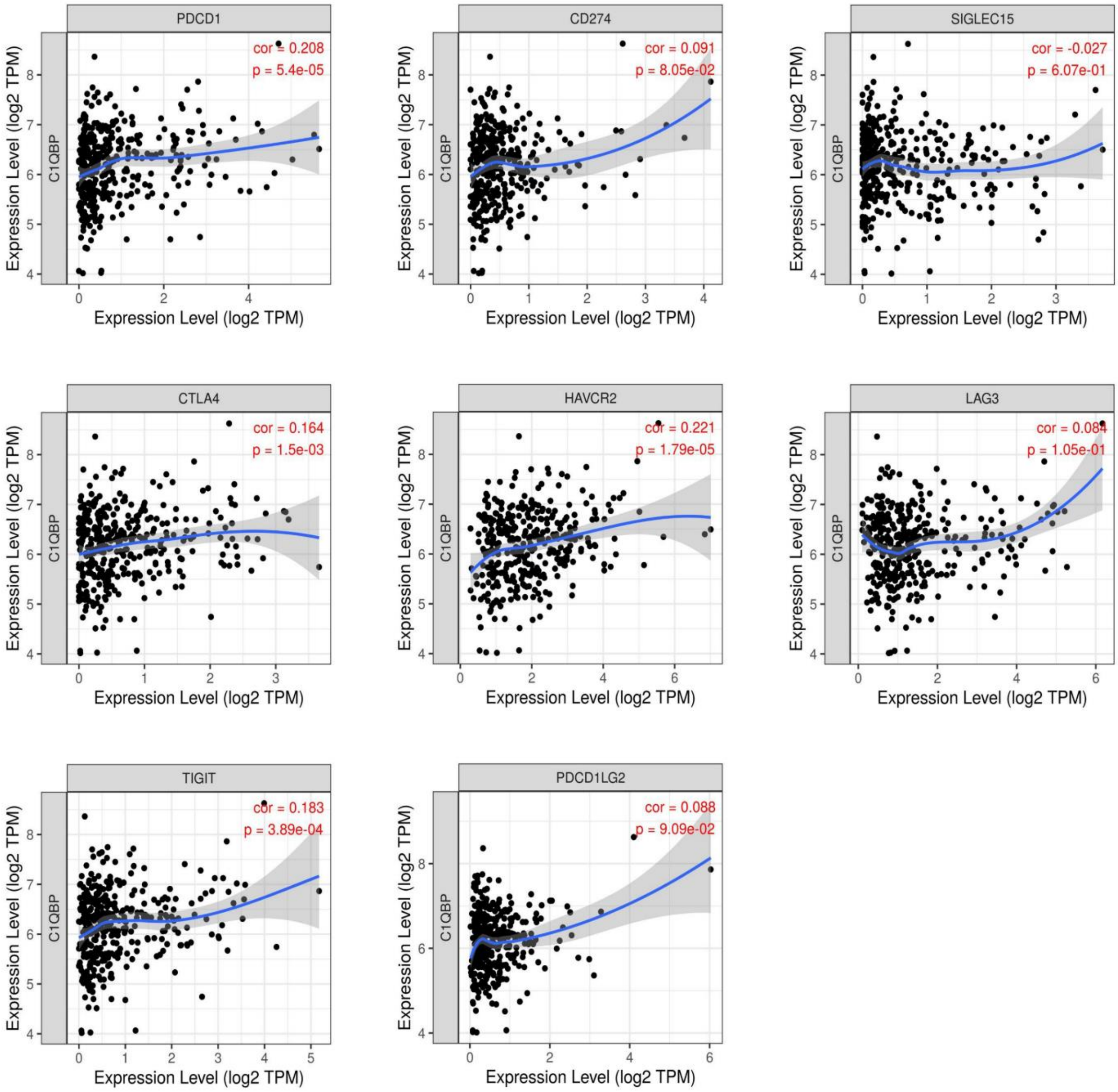

Figure S7. Correlation between *C1QBP* expression and known immune-related gene expression

Plots showing the levels of *C1QBP* mRNA and expression of various known immune-related genes by the TIMER platform.
